# Sanatorium Buildings at £12 per Bed

**Published:** 1916-11-25

**Authors:** Frank Clifford

**Affiliations:** Tuberculosis Physician. Swansea


*To the Editor of* THE HOSPITAL.

### Sanatorium Buildings at £12 per Bed.

*To the Editor of* THE HOSPITAL.

SIR,—After reading your report in the current issue, I think that I should give you further information, as my report to the Swansea Sanatorium Committee might be misunderstood.

The Australian Hospital at Haresfield is a temporary building, which, apart from the administrative block, cost £12 per bed. At Swansea we already have the land, but it is on a slope, our idea being tier after tier facing south. I consider that we could put up a temporary building on the lines of the Australian Hospital, and I put my estimate at £30 per bed, the extra £18 being for administration block with special foundation on account of the sloping ground. I must say that my committee considered that £40 might be nearer the mark, but even at that figure a building lasting about fifteen years is cheap compared with the old brick palaces, and as far as the patient is concerned the former is quite as efficacious.—Yours faithfully, FRANK CLIFFORD,

Swansea, Nov. 20, 1916. Tuberculosis Physician.

